# Cell‐free DNA fetal fraction in twin gestations in single‐nucleotide polymorphism‐based noninvasive prenatal screening

**DOI:** 10.1002/pd.5609

**Published:** 2019-11-21

**Authors:** Herman Hedriana, Kimberly Martin, Daniel Saltzman, Paul Billings, Zachary Demko, Peter Benn

**Affiliations:** ^1^ Division of Maternal‐Fetal Medicine, Department of Obstetrics and Gynecology University of California Davis Health Sacramento CA; ^2^ Natera, Inc San Carlos CA; ^3^ Icahn School of Medicine at Mount Sinai New York NY; ^4^ Department of Genetics and Genome Sciences UConn Health Farmington CT

## Abstract

**Objectives:**

The performance of noninvasive prenatal screening (NIPS) for fetal aneuploidy in twin pregnancies is dependent on the amount of placentally derived cell‐free DNA, the “fetal fraction (FF),” present in maternal plasma. We report FF values in monozygotic (MZ) and dizygotic (DZ) pregnancies.

**Methods:**

We reviewed FF in pregnancies at 10 to 20 completed weeks gestational age based on single‐nucleotide polymorphism (SNP)‐based NIPS where zygosity was routinely established in twin pregnancies. The cohort included 121 446 (96.3%) singleton, 1454 (1.2%) MZ, and 3161 (2.5%) DZ pregnancies. For DZ twins, individual FFs were measured.

**Results:**

Combined FF for DZ and MZ fetuses were 35% and 26% greater than singletons, respectively. The individual FF contributions from each fetus in DZ twins were, on average, 32% less than singletons. FF in DZ twin pairs were moderately correlated (Pearson correlation coefficient.66). When a threshold of 2.8% FF was applied to define uninterpretable results, 1.7% (2102/121 446) of singletons, 0.8% (11/1454) of MZ pairs, and 5.6% (178/3161) of DZ pairs were uninterpretable.

**Conclusion:**

For optimal aneuploidy NIPS in twin pregnancies, zygosity should be established and in DZ twins FF for both fetuses should be determined to identify those cases where results can be reliably interpreted.

## Abstract 2

What's already known about this topic?

- Adequate cell‐free fetal DNA (fetal fraction [FF]) is essential for noninvasive prenatal screening.
- FFs in twin pregnancies may be higher or lower than that found in singleton pregnancies.

What does this study add?

- In this large series of twin pregnancies, the average total FF was higher than for singletons but the per fetus FF was lower.
- There can be large differences in the two FFs in dizygotic twin pregnancies.
- Optimal prenatal aneuploidy screening in twin pregnancies requires information on both zygosity and the individual FFs.

## 1 INTRODUCTION

Utilization of cell‐free fetal DNA (cfDNA) in maternal plasma for noninvasive prenatal screening (NIPS) for fetal aneuploidy has grown rapidly and has changed obstetric care.^1^ Following its introduction as a test for fetal Down syndrome in singleton pregnancies, testing was expanded to include trisomy 18, trisomy 13, sex chromosome abnormalities, a select group of microdeletion syndromes, and can be carried out in twin pregnancies.^2^

The fetal fraction (FF), that is, the fraction of placenta‐derived cfDNA, relative to the total amount of cfDNA in maternal circulation, is correlated with NIPS performance.^3^ The accurate quantification of FF is of particular relevance in affected dizygotic (DZ) twin pregnancies where, usually, only one of the two fetuses are affected and the FF contributions from each pregnancy may not be equal. Canick et al estimated that the average combined amount of FF in twin pregnancies was 35% higher than that in singletons, implying that the average FF for each individual twin is about 2/3 as much as in singleton pregnancies.^3^ Struble et al^4^ found that monozygotic (MZ) twins had a median FF of 14% which can be compared with 13% for singletons measured using the same method.^5^ The median for the twin with the lower FF in DZ twins was 7.9%.^4^ To ensure that a chromosomally abnormal twin would nearly always be detected, setting a minimum requirement for the lower FF was proposed when determining whether the test could be interpreted. Using the same methodology, Sarno et al^6^ reported that twin pregnancies (DZ and MZ) had a median FF of 8% compared with 11% for singletons. When 4% was used as the minimum FF required, the rate of noninformative results (no‐call rate) was 9% to 11%.^6^, ^7^ Detailed protocols and no‐call rates have not been reported for other counting‐based NIPS methodologies where the individual FFs for each twin are not separately determined.

The recent availability of a single‐nucleotide polymorphism (SNP)‐based NIPS to distinguish DZ and MZ twins and measure the individual FFs in DZ pregnancies^8^ provides an opportunity to further characterize the distribution of FF in MZ and DZ twins. The objective of this study was to provide this information based on a large cohort of DZ and MZ twin pregnancies.

**What's already known about this topic?**

- Adequate cell‐free fetal DNA (fetal fraction [FF]) is essential for noninvasive prenatal screening.
- FFs in twin pregnancies may be higher or lower than that found in singleton pregnancies.

**What does this study add?**

- In this large series of twin pregnancies, the average total FF was higher than for singletons but the per fetus FF was lower.
- There can be large differences in the two FFs in dizygotic twin pregnancies.
- Optimal prenatal aneuploidy screening in twin pregnancies requires information on both zygosity and the individual FFs.

## 2 METHODS

FF data were collected for SNP‐based NIPS performed between 1 October 2017 and 30 April 2018. Plasma samples were analyzed at a Clinical Laboratory Improvement Act (CLIA)‐certified and College of American Pathologists (CAP)‐accredited laboratory (Natera, Inc; San Carlos, CA) using a previously published SNP‐based NIPS methodology.^9^

Presence or absence of twins was based on information provided by the referring physician. In rare instances, the SNP analysis indicated an undocumented DZ twin pregnancy, and these cases were included only if confirmed by the referring physician. Testing was not offered when there was a known fetal death prior to the time of NIPS. Zygosity assessment was performed by identifying the number of genetically distinct individuals represented in the maternal plasma sample; two distinct sources indicated MZ twins (one pair of genetically identical twins and the mother) while three distinct sources indicated DZ twins (two genetically distinct twins and the mother).^8^ FF was estimated using over 13 000 SNP loci on targeted chromosomes for singleton and twin fetuses based on the minor allele frequencies: specifically, a combined FF for MZ twins because they are genetically identical, and two distinct FF estimates for DZ twins. For the purpose of this descriptive study, FF were collated for MZ, DZ, and singleton pregnancies, in addition to the inclusion of maternal age, gestational age, and maternal weight at the time of NIPS blood draw. Excluded were all data with absent FF for causes of inadequate sample, incomplete referral information or uninterpretable for reasons unrelated to FF, and FF less than 1% (the limit required for reliable measurement and to exclude other atypical situations such as samples from nonpregnant women and gross gestational age errors). Although the testing was available from nine completed weeks gestational age, for the purposes of this study, we limited the analyses of FF to the time when most screening is conducted, 10 to 20 completed weeks gestational age. Specific clinical information including interpretation of the results was not abstracted. Summed and individual FFs for MZ, DZ, and singleton (S) pregnancies were compared.

Where appropriate, data evaluation included descriptive statistics, two‐sided *t*‐test, and one‐way analysis of variance (ANOVA) at the 5% significance level. No Bonferroni correction for multiple tests was applied to minimize type II (false negative) associations.

The study was based on retrospective analysis of deidentified data and therefore fell under an IRB exemption protocol, as determined by Ethical & Independent Review Services, Corte Madera, CA (E&I) protocol #: 17113‐02.

## 3 RESULTS

In the 6‐month period, 4615 twin and 121 446 singleton pregnancy test referrals met the inclusion criteria. Of the 4615 twin pairs, 1454 (31.51%) were MZ and 3161 (68.49%) were DZ.

**Figure 1:**
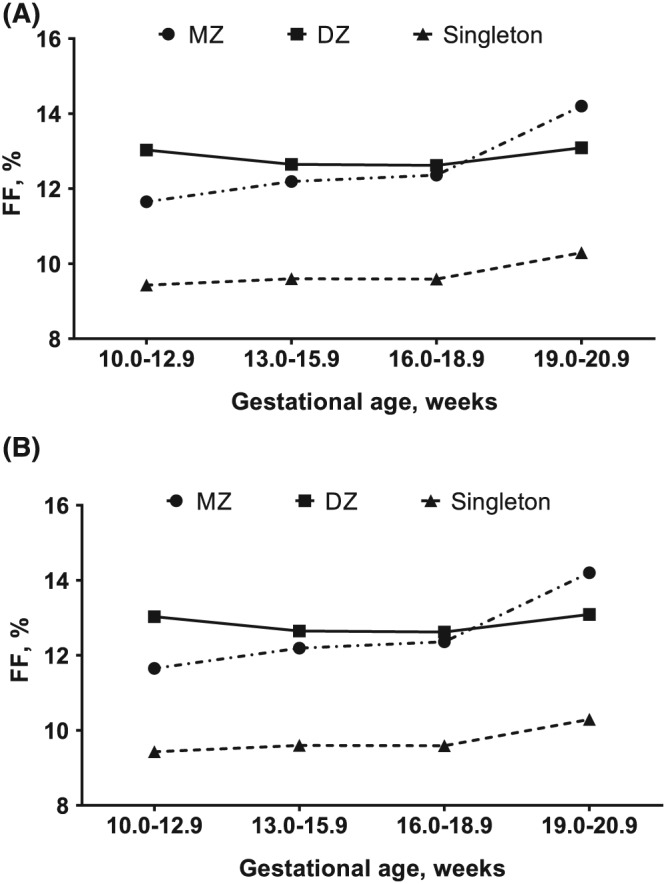
Mean fetal fraction (FF) in singletons and twins according to gestational age and maternal weight. A, Gestational age and B, maternal weight

The demographics are presented in Table 1. There were small but statistically significant differences in gestational ages when blood was drawn for NIPS for MZ, DZ, and singleton pregnancies (*P* < .0001). As expected, maternal age was somewhat higher in DZ pregnancies compared with MZ and singleton pregnancies (*P* < .0001). Women with DZ pregnancies were also significantly heavier than women with MZ or singleton pregnancies (*P* < .0001). Figure 1A shows the relationship between FF and gestational age in twin pregnancies; the increase in FF with gestational age was very similar in MZ and DZ pregnancies and parallels that seen in singletons. The relationship between FF and maternal weight was also proportionate in each of these three groups of pregnancies (Figure 1B).

**Table 1:** Distribution of gestational age, maternal age, and maternal weight in singletons and twins

Table 1. Demographics	Singleton (n = 121,446)	MZ (n = 1,454)	DZ (n = 3,161)	*P*
GA (days)				All group comparison <0.0001
Mean ± SD	90.4 ± 17.1	92.2 ± 17.9	91.9 ± 13.5	
Median	86	87	87	
Maximum	146	145	146	
Minimum	70	70	70	
MA (years)				DZ vs singleton <0.0001
Mean ± SD	31.7 ± 6.0	31.9 ± 5.7	32.9 ± 5.1	
Median	32	32	34	
Maximum	57	45	47	
Minimum	12	14	16	
MW (pounds)lbs				DZ vs singleton <0.0001
Mean ± SD	160.0 ± 42.3	160.7 ± 43.5	165.3 ± 43.2	
Median	150	150	155	
Maximum	510	363	402	
Minimum	75	93	83	

*Note*. *P* < .05 considered statistically significant.

Abbreviations: DZ, dizygotic; GA, gestational age; MA, maternal age; MW, maternal weight; MZ, monozygotic.

Table 2 summarizes the FFs in MZ, DZ, and singleton pregnancies and their statistical comparisons. Combined FF was significantly lower in MZ twin pregnancies, compared with DZ pregnancies, and higher than in singletons. In DZ pregnancies, the average individual FF (6.4%) was about 68% of that seen in singletons (9.5%). Individual FF values in MZ pregnancies are not reported because the two contributions cannot be distinguished.

**Table 2:** Differences in FF between singleton and twin groups

Fetal Fraction	Mean ± SD	Mean ± SD	Difference (%)^a^	*P*
MZ + DZ (combined FF) vs. singleton	12.6 ± 4.8	9.5 ± 4.1	+3.1 (32.4)	<0.0001
MZ vs. DZ (combined FF)	12.0 ± 5.0	12.9 ± 4.7	−0.9 (7.0)	<0.0001
MZ (combined FF) vs. singleton	12.0 ± 5.0	9.5 ± 4.1	+2.5 (25.9)	<0.0001
DZ (combined FF) vs. singleton	12.9 ± 4.7	9.5 ± 4.1	+3.4 (35.4)	<0.0001
DZ (individual FF) vs. singleton	6.4 ± 2.6	9.5 ± 4.1	−3.1 (32.3)	<0.0001

Abbreviations: FF, fetal fraction; DZ, dizygotic; MZ, monozygotic; DZ (both), the sum of the two fetal fractions.

a % Differences expressed relative to singleton (except MZ vs DZ expressed relative to MZ).

Figure S1 shows the distribution of individual FF values found in DZ pregnancies. In some laboratories, a FF cutoff of 4% has been used to define the minimum proportion of fetal DNA required for an informative result. When this cutoff was applied to the study population, 6.0% (7283/121 446) singletons, 3.7% (54/1454) MZ twin pairs, and 22.0% (695/3161) of DZ pairs had at least one fetus with FF less than 4%. For individual DZ fetuses, 15.9% (1007/6322) were associated with a FF less than 4%. The proportion of DZ pregnancies where both FFs was less than 4% was 9.9% (313/3161). Table 3 summarizes the proportions of DZ cases where one, or both, FFs were below the limit of reliable interpretation for various levels of total FF.

**Table 3:** The proportions of dizygotic pregnancies where one, or both, fetal fractions (FFs) were below the limit of reliable interpretation sub‐classified by levels of total FF.

Total FF	No. of Pregnancies	One Fetus < 4%	Two Fetuses < 4%	One Fetus < 2.8%	Two Fetuses < 2.8%
≥10%	2,215	63 (2.8%)	0 (0%)	19 (0.9%)	0 (0%)
≥8% to 10%	480	166 (34.6%)	0 (0%)	11 (2.3%)	0 (0%)
≥6% to 8%	359	151 (42.1%)	208 (57.9%)	54 (15.0%)	0 (0%)
≥4% to 6%	100	2 (2%)	98 (98.0%)	63 (63.0%)	24 (24.0%)
≥2% to 4%	7	0 (0%)	7 (100%)	0 (0%)	7 (100%)
Any	3,161	382 (12.1%)	313 (9.9%)	147 (4.7%)	31 (1.0%)

Similarly, when a threshold of 2.8% FF was applied to define uninterpretable results, 1.7% (2102/121 446) of singletons, 0.8% (11/1454) of MZ pairs, and 5.6% (178/3161) of DZ pairs had at least one fetus with FF less than 2.8%. Both FFs were below 2.8% in 1% of DZ pairs (31/3161). The proportion of individual DZ fetuses that had a FF less than 2.8% was 3.3% (209/6322).

We evaluated the impact of not measuring FF on the trisomy 21 detection rate in DZ pregnancies for NIPS protocols that cannot identify trisomy when the individual FF is either less than 4% or less than 2.8%. We assumed (a) essentially all DZ affected pregnancies have only one affected fetus, (b) that distribution of FFs from trisomy 21 fetuses are the same as those for unaffected fetuses, (c) and all fetuses with FFs below the cutoff are reported as unaffected. In this situation, all affected pregnancies where both fetuses have FFs below the cutoff would not be detected, and 50% of affected pregnancies where one fetus is below the cutoff would not be detected and estimated losses in detection rates would be likely upper limits. For these two FF cutoffs, the reduction in the detection rate would be approximately 16% and 3.4%, respectively. For a protocol where only the combined FF from both fetuses is measured and it is assumed that the individual FF contributions are equal (combined FF cutoffs greater than 8% or greater than 5.6%), 50% of affected pregnancies with one fetus below the individual FF cutoff of 4% or 2.8% would be missed. This would correspond to a reduction in the detection rate of approximately 3.6% and 1.6%, respectively.

We also considered whether the two FFs for DZ twin pairs were correlated. Figure S2 shows a plot of the paired FFs when each twin pair is randomly assigned as twin A or twin B. The Pearson linear correlation coefficient, *R*, was.66 (probability that the correlation was not significantly different from 0, *P* < .0001).

## 4 DISCUSSION

Our results show that average FF for twin pregnancies are 32% higher than for singletons. However, this difference is dependent on zygosity; combined FF for DZ and MZ fetuses are 35% and 26% greater than singletons, respectively. The individual FF contributions from each fetus in DZ twins are, on average, 32% less than singletons.

These observations have relevance to the way in which NIPS for fetal aneuploidy should be interpreted in twin pregnancies. Except in highly exceptional cases, MZ twins will be identical with respect to presence or absence of fetal aneuploidy and the fact that FF is higher than in singletons means that test performance and no‐result rate should be better than that for singleton pregnancies. However, for affected DZ twins, by far the most likely possibility is that only one of the two fetuses will be affected. Thus, NIPS in the case of DZ twins requires successful analysis of cfDNA where an affected fetus is contributing, on average, only 67% of the abnormal DNA that would be seen in an affected singleton pregnancy.

It is important to measure both individual twin FFs because there are many instances where the two FFs in a DZ pair are discrepant and measuring only the total FF will be insufficient for reliable test interpretation. Figure S2 shows numerous cases (in the upper left and lower right quadrants) where one fetus has a high FF and the other a very low FF. The reason why there are large differences in FF in some DZ pregnancies is unknown, and this merits additional research. Some cases with very low FF might be attributable to unrecorded fetal deaths or triploidy, trisomy 18 or trisomy 13.^10^

Based on a 2.8% minimum FF, we estimated that 5.6% of DZ pregnancies should not be interpreted due to low individual fetus FF but this increases to 22.0% at a 4% FF requirement. This large difference reflects the high number of individual DZ fetuses that are contributing relatively low FFs (Figure S1). Previous studies have also noted that relatively high proportions of DZ pregnancies with FF below 4%, although the proportions were not as high as those reported here.^6^, ^7^ Differences in the reported rates of cases with low FF may be due to referral population differences and the lack of standardization in FF measurements. High numbers of cases with insufficient FF for reliable test interpretation will diminish the net detection rate of affected pregnancies. Our estimates of projected loss of detection in DZ pregnancies emphasize the importance of accurately measuring FF and routinely reporting this information.

A low total FF may be interpretable for a MZ twin pregnancy but not for a DZ pregnancy. Simultaneous assessment of zygosity thus allows improved discrimination between reportable and nonreportable cases. Another benefit of assessing zygosity at the time of NIPS is that prior risks for aneuploidy are different depending on zygosity, and the correct per pregnancy prior risk can be incorporated into the risk calculation.^11^, ^12^

Although based on a large dataset, this study comes with limitations. There are multiple clinical cofactors or correlations with FF, and these were not fully considered. This includes gestational age, maternal weight, maternal serum analytes, use of assisted reproductive technology, aneuploidy, pregnancy complications, or medications that can affect FF.^13^, ^14^, ^15^ In twin pregnancies, we observed associations between FF with gestational age and with maternal weight (Figure 1A,B) that paralleled observations in singleton pregnancies. We also noted that maternal age in DZ pregnancies were significantly greater, which may be a result from multiple large developing follicles associated with advancing maternal age resulting in increasing DZ twinning in these women.^16^ Increasing maternal weight and body mass index have been associated with DZ twinning,^17^ consistent with our findings. We did not consider the extent to which assisted reproductive technology may have been used and that may further explain the finding of increased maternal weight for DZ cases because these women may be older.^14^, ^17^ We also did not distinguish between test‐positive and test‐negative cases or evaluate detection rates and false‐positive rates. It is also possible that some MZ pregnancies were not identified or reported to the laboratory and these would remain unrecognized by the laboratory testing.

In summary, because (a) FF differ between MZ and DZ pregnancies, (b) the level of FF needed for reliable testing will differ depending on zygosity, and (c) the prior risk used in screening algorithms differs depending on zygosity, it is advantageous to carefully consider both FF and zygosity at the time of NIPS. Accurate measurement of the individual FFs for DZ pregnancies will identify those cases where a high confidence result can be provided.

## CONFLICTS OF INTEREST

Zachary Demko and Paul Billings are employees of Natera, Inc and hold stock in the company. Kimberly Martin and Peter Benn are consultants to Natera and hold stock options. Herman Hedriana is a former employee of Natera and does not own stock or hold stock options. Daniel Salzman serves on the advisory board of Natera; he is not an employee and does not receive any financial compensation from Natera.

## FUNDING SOURCES

This work was funded by Natera, Inc.

## Supporting information

Data S1. Supporting Information

Data S2. Supporting Information

## Data Availability

The data that support the findings of this study are available from Dr Z. Demko (zdemko@natera.com) upon reasonable request.
