# Supplementary figures and images for: Cell‐free DNA fetal fraction in twin gestations in single‐nucleotide polymorphism‐based noninvasive prenatal screening
Source: Prenat Diagn. 2019 Nov 21;40(2):179–84. doi: 10.1002/pd.5609 (PMC7027570; doi:10.1002/pd.5609)

Supplement Figure 1

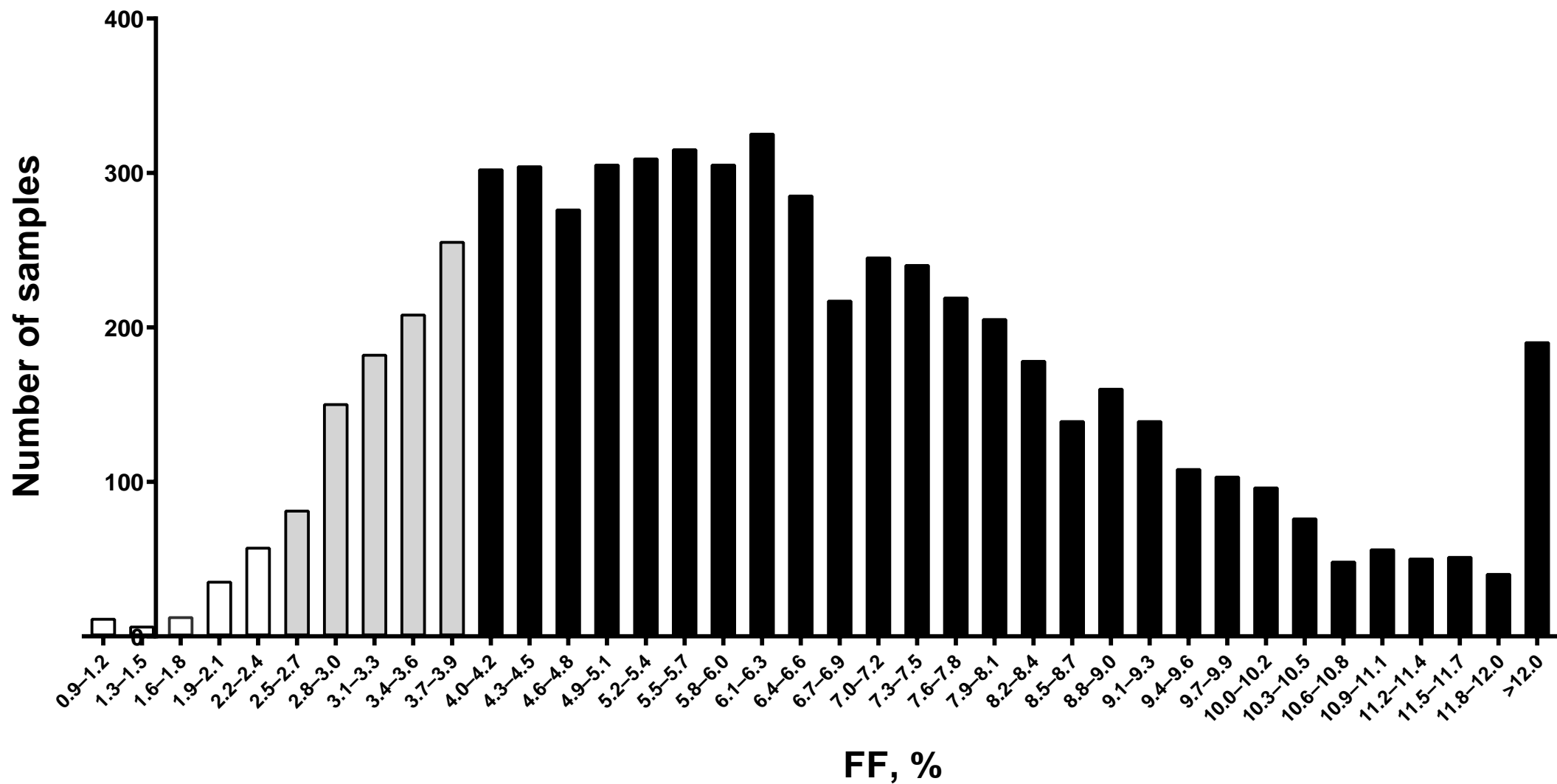

Supplement: Supplementary file 1 — Data S1. Supporting Information [file PD-40-179-s001.pdf]

**Supplement Figure 2**

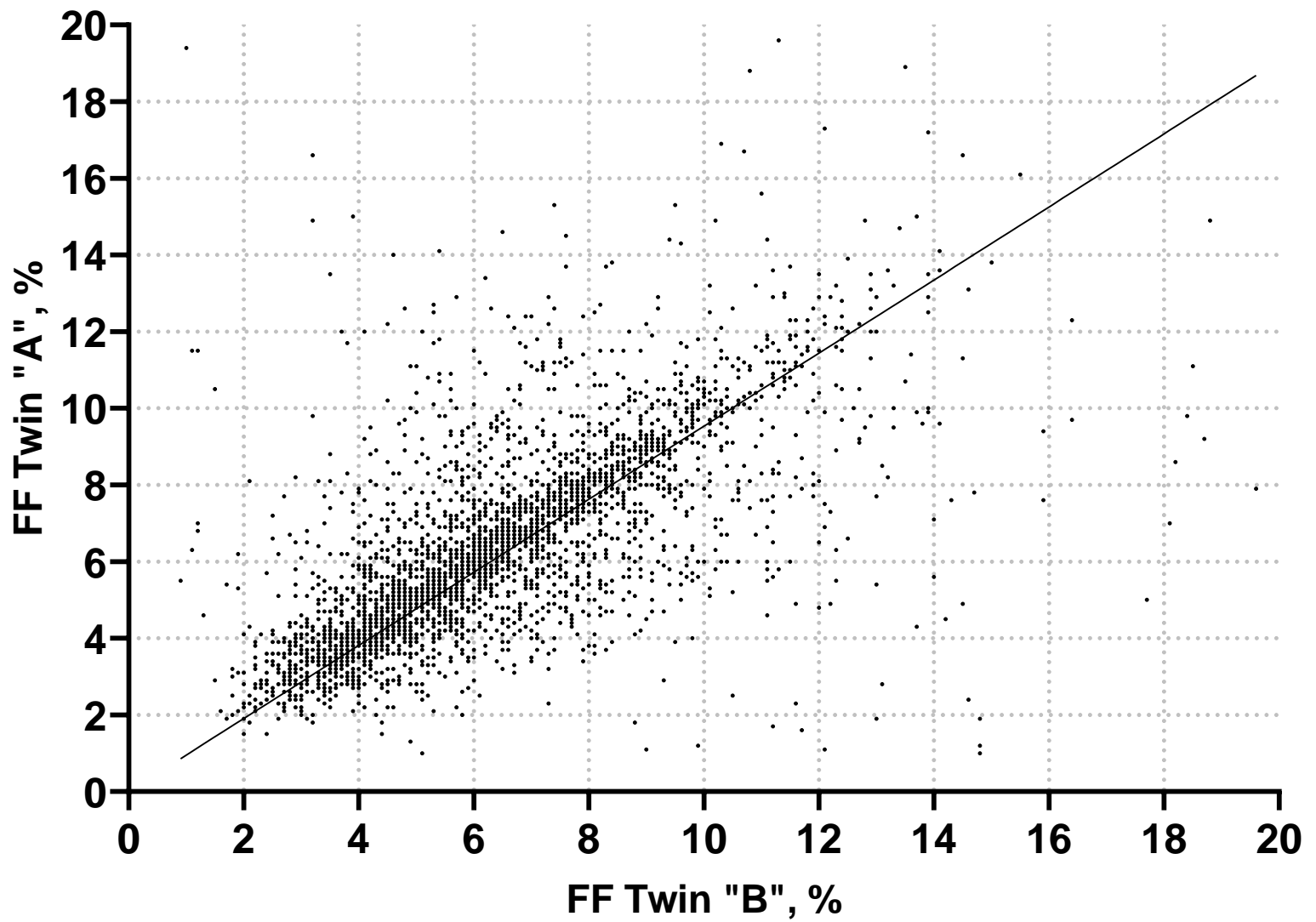

Supplement: Supplementary file 2 — Data S2. Supporting Information [file PD-40-179-s002.pdf]
